# Phospholipase Cγ1 (PLCG1) overexpression is associated with tumor growth and poor survival in *IDH* wild-type lower-grade gliomas in adult patients

**DOI:** 10.1038/s41374-021-00682-7

**Published:** 2021-10-25

**Authors:** Tianwen Li, Zhipeng Yang, Haoyuan Li, Jingjing Zhu, Ye Wang, Qisheng Tang, Zhifeng Shi

**Affiliations:** 1 Department of Neurosurgery, Huashan Hospital, Fudan University, Shanghai, China; 2 National Center for Neurological Disorders, Shanghai, China; 3 Institute of Neurosurgery, Fudan University, Shanghai, China; 4 Institute of Engineering, Fudan University, Shanghai, China; 5 Department of Pathology, Huashan Hospital, Fudan University, Shanghai, China; 6 Shanghai Key Laboratory of Neural Regeneration and Brain Function Restoration, Shanghai, China

**Keywords:** CNS cancer, Cancer epigenetics, Genetic markers

## Abstract

Gliomas are the most common and recalcitrant intracranial tumors, approximately a quarter of which are classified as lower-grade gliomas (WHO II–III). Although the prognosis of lower-grade gliomas (LGGs) is significantly better than that of higher-grade gliomas, as a highly heterogeneous tumor type, the prognosis of LGGs varies greatly based on the molecular diagnosis. IDH wild-type used to be regarded as a dismal prognostic biomarker in LGGs; however, several studies revealed that IDH wild-type LGGs might not always be equivalent to glioblastoma (WHO IV). Hence, we hypothesize that underlying biological events in LGGs can result in different prognosis. In our study, transcriptome profiling was performed in 24 samples of LGG, and the results showed that the expression of phospholipase Cγ1 (PLCG1) was significantly correlated with IDH1/2 status and patients’ clinical outcome. Furthermore, the cancer genome atlas (TCGA) and the Chinese glioma genome atlas (CGGA) databases verified that elevated PLCG1 expression was associated with tumor progression and poor survival in LGG patients. Moreover, PLCG1-targeted siRNA dramatically affected the growth, migration and invasiveness of IDH wild-type LGG cell lines. In in vitro and in vivo experiments, the PLC-targeted drug significantly suppressed the tumor growth of IDH wild-type LGG cell lines in vitro and tumors in mouse models. Taken together, our results demonstrated that higher PLCG1 expression was associated with tumor growth and worse prognosis in IDH wild-type LGGs and PLCG1 could serve as a potential therapeutic target for IDH wild-type LGG patients.

## Abstract (web summary)

The mRNA expression levels of phospholipase Cγ1 (PLCG1) are much higher in IDH wild-type (IDHwt) lower-grade gliomas (LGGs) than in that of IDH mutant (IDHmut) LGGs. Higher PLCG1expression in IDHwt LGGs indicates poor clinical outcome. PLCG1 amplification may act as the key mechanism of PLCG1 upregulation. Depletion of PLCG1 expression can inhibits proliferation and invasion of IDHwt LGG cell lines in vitro and in vivo. The PLC inhibitor U73122 may therefore be a potential therapeutical agent for IDHwt LGGs.

## Introduction

Glioma is the most common malignant tumor of the central nervous system, with an increasing incidence rate in recent years^1^. According to the WHO 2021 classification system, adult diffuse gliomas can be defined as “astrocytoma, IDH-mutant”, “oligodendroglioma, IDH-mutant, 1p19q-codeletion” or “glioblastoma, IDH-wildtype”^2^. Typically, the first two categories are regarded as “lower grade gliomas”, while glioblastoma is considered a “higher grade glioma”. However, despite the aggressive nature of IDH mutant (IDHmut) LGGs, ~20–25% of LGGs are characterized by IDH wild-type (IDHwt)^2–5^. IDHwt LGG patients were older, had fewer frontal lobe lesions and had a poor prognosis, with a 5-year survival rate of 20%, compared to their IDHmut counterparts^6–8^. Recently, several studies on IDHwt LGG revealed high genetic heterogeneity and different clinical outcomes in this tumor subtype^9,10^. cIMPACT-NOW update 3 has refined the definition of IDHwt LGGs and they recommended that IDHwt LGG with EGFR amplification or TERTp mutation or combined chromosome +7/−10 can be diagnosed as “diffuse astrocytic glioma, IDHwt with molecular features of glioblastoma, WHO grade IV”^11,12^. Meanwhile, in the recent publications, histological grade and comprehensive molecular assessment were reported to be significantly important for clinical stratification in IDHwt LGGs with molecular features of glioblastoma, especially for TERT promoter mutation status and methylome characteristics^13,14^. Large-scale deep sequencing based on the original specimens from The Cancer Genome Atlas (TCGA) was conducted and stratified IDHwt LGGs into four subtypes: classic-like, mesenchymal-like, LGm6-GBM and PA-like^15^. All these findings indicated that different biological pathways might be involved in IDHwt glioma oncogenesis and malignant transformation, especially transcriptome and post-transcription alterations in IDHwt LGG, which need to be further elucidated.

In our earlier research, we conducted transcriptome profiling of 24 LGG samples (5 IDHwt and 19 IDHmut LGGs). On chromosome 20, we found a candidate gene, PLCG1, that was overexpressed in IDHwt LGGs. PLCG1 is located on chromosome 20q12-13.1 and encodes phospholipase Cγ, an enzyme that hydrolyzes phosphatidylinositol-4,5-bisphosphate (PtdIns(4,5)P2) into the second messengers diacylglycerol (DAG) and inositol-1,4,5-trisphosphate (IP3) using calcium as a cofactor. These molecules can in turn activates a number of signaling pathways^16^. PLCG1 can be found in various subcellular compartments, including the cytosol and plasma membrane^16,17^. This gene participates in the regulation of migration, invasion, and cell spreading^18,19^. Recurrent PLCG1 mutations that cause constitutive activation of the protein have been described^20,21^. Furthermore, it plays an important role in the regulation of intracellular signaling cascades and intracellular transduction of receptor-mediated tyrosine kinase activators among various tumors^18,19,22–24^, including glioblastoma^22^. However, the biological effects of PLCG1 overexpression in IDHwt LGGs remain unclear. Herein, we aimed to study the mechanism and clinical significance of PLCG1 in IDHwt LGGs and explore its potential therapeutic role in treating IDHwt LGGs.

## Materials and methods

### Patients and specimens

Our study was approved by The Ethics Committee of Huashan Hospital, Fudan University, and all experiments complied with the current laws of PR China. All IDHwt LGG tumor specimens were obtained from the Huashan Glioma Biobank^25,26^. All patients who provided a specimen in the Huashan Glioma Biobank provided consent, and the samples were collected during surgery, snap frozen in liquid nitrogen and fixed with formalin in preparation for paraffin blocking. The diagnoses of all gliomas were based on the WHO Classification of Central Nervous System Tumors 2016. IDH status, medical history and clinical follow-up data were all retrieved from the Biobank E-system.

### TCGA and CGGA data retrieval

The data from the TCGA and CGGA validation cohorts were retrieved from the “low grade glioma database” (TCGA, https://www.cancer.gov/about-nci/organization/ccg/research/structural-genomics/tcga/ and CGGA, http://www.cgga.org.cn/), and used to validate the prognostic value and function of PLCG1 in gliomas.

For LGG data from TCGA, we downloaded the Gene Expression RNAseq (https://xenabrowser.net/datapages/?dataset=TCGA-LGG.htseq_fpkm.tsv&host=https%3A%2F%2Fgdc.xenahubs.net&removeHub=https%3A%2F%2Fxena.treehouse.gi.ucsc.edu%3A443), phenotype data (https://xenabrowser.net/datapages/?dataset=TCGA-LGG.GDC_phenotype.tsv&host=https%3A%2F%2Fgdc.xenahubs.net&removeHub=https%3A%2F%2Fxena.treehouse.gi.ucsc.edu%3A443) and survival data (https://xenabrowser.net/datapages/?dataset=TCGA-LGG.survival.tsv&host=https%3A%2F%2Fgdc.xenahubs.net&removeHub=https%3A%2F%2Fxena.treehouse.gi.ucsc.edu%3A443) from Xena database (UCSF).

For LGG data from CGGA, we downloaded the “mRNAseq_693” from http://www.cgga.org.cn/download.jsp.

We established the following criteria for patient screening in these two database: ^①^WHO Grade II-III, ^②^IDH1/2 mutation status data, ^③^survival status and overall survival data. As long as the above three types of data were available, we would include these cases in this study, and there are no other exclusion criteria.

### RNA sequencing and analysis, next generation sequencing (NGS) and gene set enrichment analysis (GSEA)

Twenty-four samples from patients diagnosed as LGG (19 IDHmut and 5 IDHwt) were collected. Total RNA isolation, sequencing, and analysis were conducted by Shanghai Oebiotech Co. Ltd. (Shanghai, China). Genes were defined as differentially expressed when their logarithmic expression ratio was more than twofold (*P* < 0.05). The mRNA level was defined as differentially expressed when logarithmic expression ratios were >1.5-fold (*P < 0.05). The NGS was also conducted by Shanghai Oebiotech Co. Ltd. (Shanghai, China) according to their NGS protocol.

For GSEA of the CGGA and TCGA LGG cohorts, the raw data were downloaded, GSEA was performed by clusterProfiler (R package, V.3.4.6)24, and GSEA plots were generated with enrichrplot (R package).

### cDNA synthesis and quantitative polymerase chain reaction

We retrospectively collected 152 frozen IDHwt LGG samples from patients who underwent surgical resection and were pathologically diagnosed as lower-grade glioma in Huashan Hospital from 2013–2016. A total of 126 patients with complete clinical follow-up data were enrolled in this study (Table S1). Total RNA was collected using TRIzol reagent (In﻿vitrogen) and extracted using an miRNeasy Mini Kit (Qiagen) according to the manufacturer’s protocol. Cell line cDNA was obtained using HiScript II Q RT SuperMix for qPCR (Vazyme). Gene expression was quantified using TaqMan gene expression assays (PLCG1: Hs01008225_m1, GAPDH: Hs02758991_g1 and TBP (TATA-box binding protein): 4333769 F; Invitrogen) using an Applied Biosystems 7500 cycler (Thermo Fisher).

### Immunohistochemistry (IHC)

Twenty-two samples (11 IDHwt LGG and 11 IDHmut LGG) from the Huashan Glioma Biobank were randomly collected for IHC (Table S2). To ensure the integrity of the specimens and accuracy of IHC, these 22 samples were from patients who underwent surgical resection and diagnosed as lower grade glioma pathologically in Huashan hospital in the second half of 2016. Tissue sections were deparaffinized in xylene and rehydrated in a graded series of alcohol. Tissue slides were then immersed in boiling epitope retrieval solution (pH 6; Novocastra) for 10 min. Immunohistochemical staining was performed using an UltraVision Quanto Detection System HRP DAB (Thermo Fisher) according to the manufacturer’s protocol. Sections were incubated with a rabbit polyclonal anti-PLCG1 antibody (#5690, Cell Signaling Technology) at a 1:100 dilution in 1% bovine serum albumin (BSA)/phosphate-buffered saline (PBS) at room temperature for 1 h. All sections were counterstained with haematoxylin (Sigma) and mounted with mounting medium. To score the IHC staining data, semi-quantitative scoring system which considered the percentage of PLCG1-positive cells and the staining intensity was employed to determine the PLCG1 expression. The percentage of PLCG1-stained tumor cells was graded into 0 (0%), 1 (1–10%), 2 (11–50%), 3 (51–80%), 4 (>80%). The staining intensity was graded into 0 (negative), 1 + (weak), 2 + (moderate), and 3 + (strong). A final score was obtained by multiplying the score for percentage of stained cells with the score for staining intensity. Six images of each sample were taken, and the average integrated optical density was measured by ImageJ software ﻿(National Institutes of Health). IHC staining for PLCG1 was scored according to standard criteria as 0, 1, 2, 3 or 3+, where 0–3 was considered negative and 3+ was considered positive.

### Fluorescence in situ hybridization (FISH) and TCGA copy number variation (CNV) analysis

The same case cohort from IHC testing was enrolled for FISH assay (Table S2). FISH assays were utilized to detect the amplification of PLCG1 in glioma cells. Genechem Co. Ltd. (China) supplied the fluorescent PLCG1 FISH probe and the fluorescent CEP2 (CDC42EP2) FISH probe (control). LGG samples from patients were fixed with 4% paraformaldehyde, immobilized on slides and then co-incubated overnight with the FISH probes (50 nmol/L) at 37 °C. A fluorescence microscope (Olympus) was used to view the fluorescent images, which were digitally captured.

The corresponding LGG patients’ metadata was downloaded from UCSC Xena (http://xena.ucsc.edu/) and mapped based on sample IDs extracted from the TCGA barcode. Then, we downloaded the clinical data of LGG patients from the cBioportal (https://www.cbioportal.org/) database for further bioinformatic analysis. First, we extracted the CNV data of PLCG1, and the R language “ggpubr” and “ggplot2” packages was used to perform Rank Sum Test on the difference of PLCG1 copy number gain/amplification in the IDHmut/ IDHwt groups. The PLCG1 CNV in TCGA_LGG pipeline transforms a CNV value into a segment mean, where Segment mean = log_2_(CNV/2). A copy number can be derived from the segment means by calculating 2 * (2^segment mean^). With this, the diploid regions will have a segment mean value of zero, amplified regions will have positive values, and deletions will have negative values.

The PLCG1 copy number gain/amplification and mRNA level of the patients were matched by patient ID in TCGA_LGG database for subsequent Pearson correlation analysis.

### Cell culture

The LGG cell lines SW1088 and SW1783 were obtained from ATCC. All cell lines were characterized as mycoplasma free by polymerase chain reaction (PCR). The cells were cultured at 37 °C in a humidified incubator with 5% CO2 and 95% O2 in DMEM supplemented with 10% foetal bovine serum (Gibco, Waltham, MA, USA) and penicillin-streptomycin (10,000 U/mL) (Gibco, Waltham, MA, USA).

### Knockdown of PLCG1 in cell lines

PLCG1-specific siRNAs (si336, si1303 and si1384) and control vector (siCtrl) (GenePharma Co. Ltd.) were delivered into cells using Rfect infection reagent (Changzhou BaiDai Co., Ltd). Approximately 8 h after transfection, the transfection medium was replaced with fresh medium. Since si336 and si1384 had no obvious knockdown effect, we used si1303 in subsequent experiments. The siRNA sequences are listed in Table S3.

### Western blotting

Cells were treated with various concentrations of each tested compound for a designated time. Then, the cells were lysed using 1× SDS sample lysis buffer containing protease and phosphatase inhibitors. Cell lysates were loaded and electrophoresed onto 8–12% SDS-PAGE gels, and then the separated proteins were transferred to PVDF membranes, which were blocked with 5% fat-free milk in TBS solution containing 0.5% Tween-20 for 4 h at room temperature. Then, the membranes were incubated with the corresponding primary antibody (1:1000–1:200) overnight at 4 °C, after which they were washed with TBST and incubated with HRP-conjugated secondary antibody for 2 h. The protein signals were visualized by an ECL Western blotting detection kit (Thermo Scientific, Waltham. MA, USA) and detected with an Amersham Imager 600 system (GE, Boston, MA, USA). Commercial antibodies against PLCG1, beta-actin-HRP and anti-rabbit HRP secondary antibody were obtained from Cell Signaling Technology. The CDK1 and Caspase3 antibody were obtained from Abcam Technology.

### Bromodeoxyuridine (BrdU) proliferation assay, cell cycle analysis, and apoptosis detection

SW1088 and SW1783 cell suspensions (3.0 × 104 cells/ml) were plated on 35 mm confocal dishes (Biosharp). Then, bromodeoxyuridine (BrdU) (Roche) with U73122, DMSO, or PBS was added to the medium. After 48 h, cells were fixed with 70% acidic ethanol for 20 min at 4 °C, followed by incubation with primary anti-BrdU antibody (ab6326, Abcam), then Alexa-488 secondary antibody, and finally DAPI. BrdU- and DAPI-positive cells were then counted in ImageJ.

For cell cycle analysis, cells were serum starved for 24 h, and then the medium was replaced with complete growth medium. After 12 or 24 h, cells were collected for DNA staining using propidium iodide (PI), and DNA content was analysed by using a BD Canto II Flow Cytometry System (BD Biosciences) and FlowJo software, v10.6.

For the apoptosis assay, cells were serum starved as described above and then freshly collected and stained for early apoptosis markers using a fluorescein isothiocyanate (FITC) annexin V apoptosis detection kit (BD Biosciences) according to the manufacturer’s instructions. Annexin V and PI staining were measured using a flow cytometer and analysed using FlowJo v10.6.

### Cell migration assay and cell invasion assay

SW1088 and SW1783 cell suspensions (4.0 × 10^4^ cells/ml) were prepared in serum-free DMEM and seeded into the upper chamber of a Transwell insert (200 μl). The lower chamber contained 700 μl of complete medium. After continuous culture for 24 h, the membranes were fixed in 4% paraformaldehyde for 15 min followed by drying and staining with 0.5% crystal violet for 15 min at room temperature. For the invasion assay, BD Matrigel™ was added to the Transwell chambers before the cells were seeded, and the culture time was 48 h.

### Construction of SWl088/Luc cells, in vivo subcutaneous tumor growth and IHC experiment of xenograft

Packaged luciferase lentiviruses were purchased from Shanghai Bioegene Co., Ltd. Infected cells were treated with puromycin for 3 days to select a stable cell line.

Four-week-old male NOD-SCID mice with body weights ranging from 23 to 26 g were subcutaneously injected with 100 μl of Matrigel (Corning) containing 8 × 10^6^ tumor cells. Tumor sizes and the weights of the mice were measured every other day. ﻿The size of xenografts was evaluated with bioluminescence imaging (PerkinElmer, Waltham, Massachusetts, USA). Mice were euthanized 20 days after SW1088 cell implantation. All animal experiments were approved by the Institutional Animal Care and Use Committee of Fudan University.

The tumors from xenografts were collected and fixed with 4% paraformaldehyde solution and embedded in paraffin. The sliced tissues (thickness: 4 mm) mounted on the glass slides were incubation by primary antibody (PLCG1, Cell Signaling Technology) and secondary antibody (Anti-rabbit, Cell Signaling Technology), then via DAB chromogenic reaction and visualized using a digital microscope (Leica). The cell nuclei were stained with DAPI. ﻿Briefly, to score the IHC staining data, five images of each sample were taken and the average integrated optical density was measured by Image Pro Plus 6.0 software.

### Statistical analysis

All experiments were performed at least three times. The results are presented as the means ± SD by Student’s *t* test using GraphPad Prism 6.0. Survival curves were generated by Kaplan–Meier analysis. As result, we divided patients (including our own patients and patients from public database) into high- and low-expression groups by median expression of PLCG1. Survival curves and hazard ratios (HRs) were compared between groups using the log-rank test. In addition, the association between IDH status and PLCG1 expression or amplification was assessed using Fisher’s exact test or the chi-square test. All significant differences were defined as *p < 0.05, **p < 0.01, and ***p < 0.001.

## Results

### IDH-dependent alterations in the gene transcription profiles of LGGs and the association between PLCG1 and survival

**Fig. 1:**
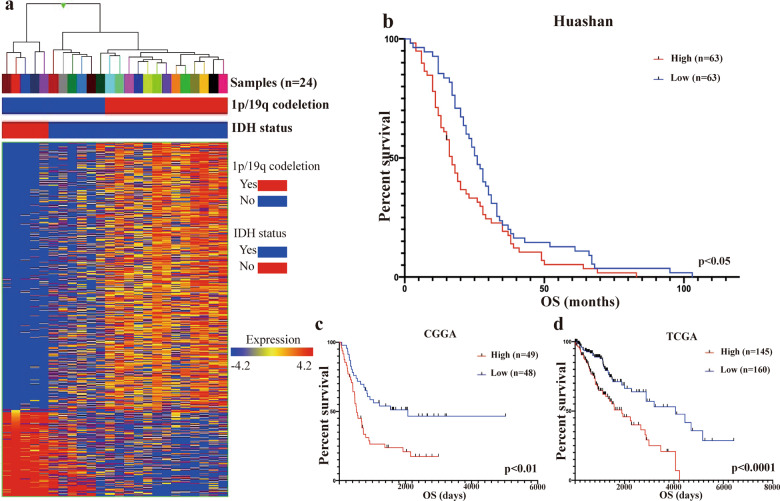
Distinct expression profiles and salience of PLCG1 between IDHwt LGG and IM LGG. **a** Hierarchical cluster analysis of 1183 highly variable genes (rows) in 24 LGG cases (columns). **b** Kaplan–Meier survival analysis of retrospective follow-up studies of IDHwt LGG patients admitted to Huashan Hospital from 2013–2016 (divided by median PLCG1 expression, *p* < 0.05). **c** Kaplan–Meier survival analysis of PLCG1 in IDHwt patients from the CGGA_LGG database (divided by median PLCG1 expression, *p* < 0.01). **d** Kaplan–Meier survival analysis of PLCG1 in IDHwt patients from the TCGA_LGG database (divided by median PLCG1 expression, *p* < 0.0001).

In our transcriptome profiling results, we identified 302 upregulated genes and 881 downregulated genes in IDHwt LGGs (Fig. 1a). On chromosome 20, we found five candidate genes, namely, COL9A3, JAG1, MRGBP, NINL, and PLCG1 (Fig. S1). Among them, PLCG1 was verified to be significantly overexpressed in IDHwt LGG compared to IDHmut LGG by TaqMan-based quantitative RT-PCR (Fig. S1a). This result is consistent with what we observed in the TCGA and CGGA cohorts (Fig. S1b, c).

PLCG1 overexpression was associated with poor clinical outcome. In our patient cohort, we identified the PLCG1 expression level and overall survival (OS) in 126 IDHwt LGG samples (Table S1), and patients with higher expression of PLCG1 had shorter survival than patients with lower expression (17 months vs. 26 months, *p* < 0.05) (Fig. 1b). Interestingly, in the CGGA_LGG database, we found that high PLCG1 expression correlated with poor outcome in IDHwt LGG patients but not in IDHmut LGG patients (*p* = 0.0587) despite with a prognostic stratification trend (Figs. 1c and S2a, b). Although PLCG1 acted as a prognostic stratifier in both IDHwt (*p* < 0.0001) and IDHmut LGG (*p* < 0.05) patients in the TCGA_LGG database, the hazard ratio of PLCG1 in IDHwt LGG was much higher than that in IDHmut LGG (19.191 vs. 4.138) (Figs. 1d and S2c, d). Such a finding was also validated in the CGGA cohorts (Figs. 1c and S2a, b). In summary, there was an undeniable trend that PLCG1 overexpression led to worse outcomes in all LGG patients, but the prognostic power of significance was obviously decreased in IDHmut patients (Figs. 1b–d and  S2).

### PLCG1 overexpression was involved in essential tumor biological processes and accompanied by gene amplification

**Fig. 2:**
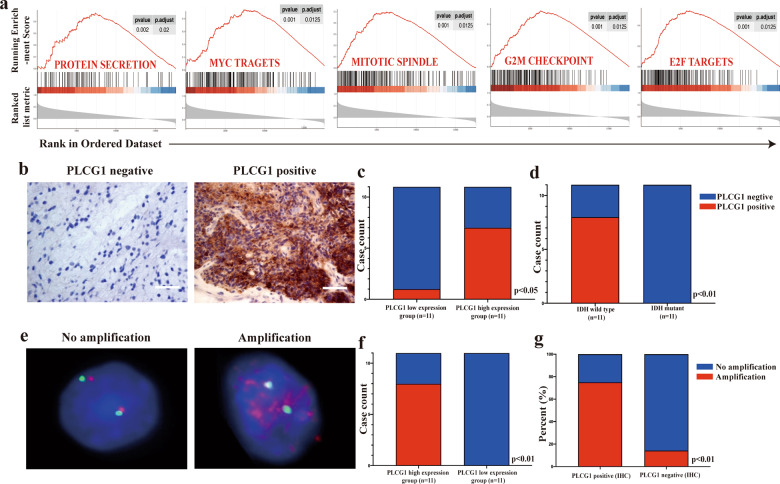
The importance of PLCG1 in IDHwt LGG and PLCG1 is associated with IDH status at the DNA and protein levels. **a** Enrichment plots of GSEA using the gene expression profiles of IDHwt LGG from the CGGA database indicated the top 5 functions that PLCG1 involved protein secretion, MYC targets, mitotic spindle, G2/M checkpoint, and E2F targets. **b** Representative photomicrographs of PLCG1 immunostaining of PLCG1-negative and PLCG1-positive samples. ﻿Scale bar = 50 µm. **c** The PLCG1 IHC score was correlated with the PLCG1 mRNA level (*p* < 0.05). **d** The PLCG1 IHC score was correlated with IDH status (*p* < 0.01). **e** Representative FISH image of nuclei of LGG cells displaying PLCG1 gene amplification and no alteration in PLCG1 copy number. PLCG1 and CEP2 (control) were labeled with red and green, respectively. CEP2, centrosome protein 2. **f** PLCG1 amplification was correlated with PLCG1 mRNA levels (*p* < 0.01). **g** PLCG1 amplification was correlated with the PLCG1 IHC score (*p* < 0.01).

To further understand the role of PLCG1 in IDHwt LGG oncogenesis, we performed GSEA to verify the associated biological processes and signaling pathways of PLCG1 on the basis of its expression level for classifying the IDHwt CGGA_LGG cohort. Overexpression of PLCG1 was correlated with five essential cancer-related processes and pathways: protein secretion, MYC targets, mitotic spindle, G2/M checkpoint, and E2F targets (Fig. 2a), which demonstrates that PLCG1 is possibly involved in the facilitation of cancer proliferation and adaptation. Consistent GSEA results were found in the IDHwt TCGA_LGG cohort (Fig. S3).

PLCG1 IHC was conducted in 11 IDHwt and 11 IDHmut LGG samples (Table S2 and Fig. 2b). As expected, 8/11 (72.73%) IDHwt LGG cases were PLCG1 positive, while no PLCG1 positive cases were present in the IDHmut group (Fig. 2d). In addition, 10/11 (91.91%) cases with high PLCG1 mRNA levels were PLCG1 positive (Fig. 2c).

To explore the mechanisms for PLCG1 dysregulation in IDHwt LGG, we employed FISH to investigate gene amplifications of PLCG1 at chromosome 20q12-13.1 in these 22 LGG samples. High PLCG1 mRNA levels were always accompanied by PLCG1 amplification (72.73%) compared with lower PLCG1 expression levels (Fig. 2e, f). To find out whether gene amplification was related to high PLCG1 protein expression, we directly compared the percent of PLCG1 amplification in PLCG1 positive group and PLCG1 negative group. Remarkably, PLCG1 amplification was strongly correlated with PLCG1 staining positivity by IHC (Table S2 and Fig. 2g). Out of the 8 IDHwt tumors with PLCG1 IHC positive staining, 6 of them (75%) displayed increased amplification of PLCG1 (Fig. 2g), suggesting that chromosomal aberration is one of the mechanisms by which PLCG1 is overexpressed (Table S2). None of the IDHmut cases showed PLCG1 amplification. The PLCG1 copy number gain/amplification analysis in TCGA_LGG database was consistent with above results which showed that PLCG1 copy number gain/amplification was much higher in IDHwt group than in that of IDHmut group (Fig. S4a). Moreover, the PLCG1 mRNA level was closely related with PLCG1 copy number gain/amplification in IDHwt group, but not in IDHmut group (Fig. S4b). Thus, there is strong evidence to support the notion that genomic abnormalities are one of the possible mechanisms for PLCG1 dysregulation in IDHwt LGG.

### Knockdown of PLCG1 affected the proliferation, migration, invasion and cell cycle of IDHwt LGG tumor cells

**Fig. 3:**
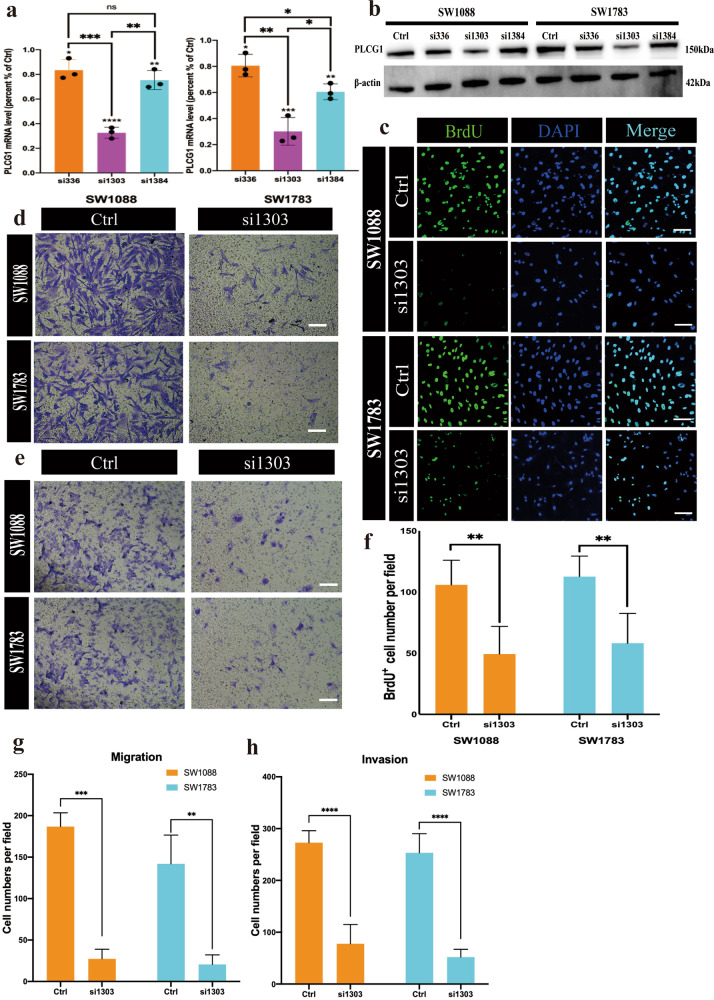
siRNA-mediated knockdown of PLCG1 affects the proliferation, migration and invasion of IDHwt LGG tumor cells. **a** Levels of PLCG1 mRNA in SW1088 and SW1783 cell lines transfected with gene-specific siRNAs (relative to those in cells with control siRNA). **b** The expression of PLCG1 protein examined in SW1088 and SW1783 cells by Western blotting. **c** Representative images of the BrdU proliferation assay reflecting the effect of siRNA on SW1088 and SW1783 cells. Scale bar = 30 µm. **d** Representative images of the migration assay reflecting the effect of siRNA on SW1088 and SW1783 cells. Scale bar = 20 µm. **e** Representative images of the invasion assay reflecting the effect of siRNA on SW1088 and SW1783 cells. Scale bar = 20 µm. **f** The statistical results of the BrdU proliferation assay. **g** The statistical results of the SW1088 and SW1783 migration assays. **h** The statistical results of the SW1088 and SW1783 invasion assays. ****p < 0.0001, ***p < 0.001, **p < 0.01, *p < 0.05. ﻿Data are expressed as the means ± SD.

To determine the important functional roles of PLCG1 in IDHwt LGG, we conducted loss-of-function studies using gene-specific siRNAs against PLCG1 in two IDHwt LGG cell lines, SW1088 and SW1783. Depletion of PLCG1 was first confirmed by quantitative qPCR and Western blot (Fig. 3a, b). siRNA-mediated knockdown of PLCG1 significantly inhibited the growth of IDHwt LGG cells, as measured by the BrdU proliferation assay (Fig. 3c, f). The number of BrdU-positive cells of si1303 group in the SW1088 and SW1738 cell lines accounted for only half of the control groups (Fig. 3f). PLCG1 knockdown also reduced the migration and invasion capabilities of IDHwt LGG cell lines in the same pattern, and much lower cell migration and poor invasion ability were observed in the si1303 group than control (Fig. 3d, e, g, h).

**Fig. 4:**
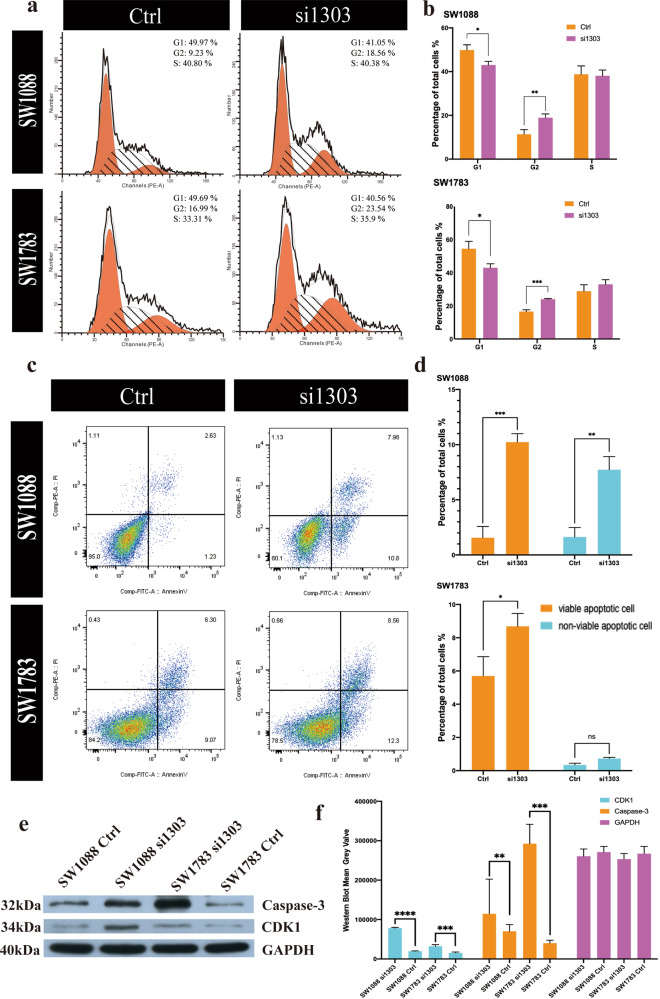
siRNA-mediated knockdown of PLCG1 arrested the cell cycle and promoted apoptosis. **a** Representative graphs showing cell cycle distribution. **b** The statistical results of the cell cycle distribution (G1, G2, and S) of SW1088 (up) and SW1783 (down) cells in the control and si1303 groups. **c** Representative graphs showing the effect of U73122 on early and late apoptosis by annexin V–PI flow cytometry assays. **d** The statistical results of the SW1088 (up) and SW1783 cell (down) apoptosis assays. **e** The Western blot verified the flow cytometry results of cell cycle and apoptosis. **f** The statistical results of the Western blot in the control and si1303 groups of CDK1 and Caspase3. ***p < 0.001, **p < 0.01, *p < 0.05. The data are expressed as the means ± SD.

In addition, flow cytometric analysis showed that the number of tumor cells arrested in G2 phase increased significantly (Fig. 4a, b), suggesting the importance of PLCG1 in the cell cycle and proliferation. Cell cycle analysis of both SW1088 and SW1783 cells showed that the number of cells in G2 phase was 1.5–2 times higher in the si1303 group than in the control groups (Fig. 4b).

GSEA (Fig. 2a) indicated that PLCG1 was involved in the pivotal process of cell viability, and we verified this finding in an apoptotic assay experiment. In the SW1088 cell line, the percentage of viable apoptotic cells was increased 4–5-fold in the si1303 group, while the percentage of nonviable apoptotic cells was increased 3–4-fold in the si1303 group. In SW1783 cells, the percentage of viable apoptotic cells was increased in the si1303 group compared with the control group. The number of nonviable apoptotic cells increased slightly in the si1303 group, with no statistical significance (Fig. 4c, d).

Then, we employed cyclin-dependent kinase 1 (CDK1, an important marker in cell cycle regulation and apoptosis) and Caspase-3 (an essential marker in apoptosis) to verify the results of flow flow-cytometric analysis. The Western blot results were consistent with flow-cytometric results of both apoptosis and cell cycle (Fig. 4e, f).

All these data suggest the biological effects of PLCG1 upregulation in regulating oncogene-like properties such as growth, migration, and invasion of IDHwt LGG cells.

### U73122 suppressed tumor growth and promoted apoptosis of IDHwt LGG cells in vitro and in vivo

**Fig. 5:**
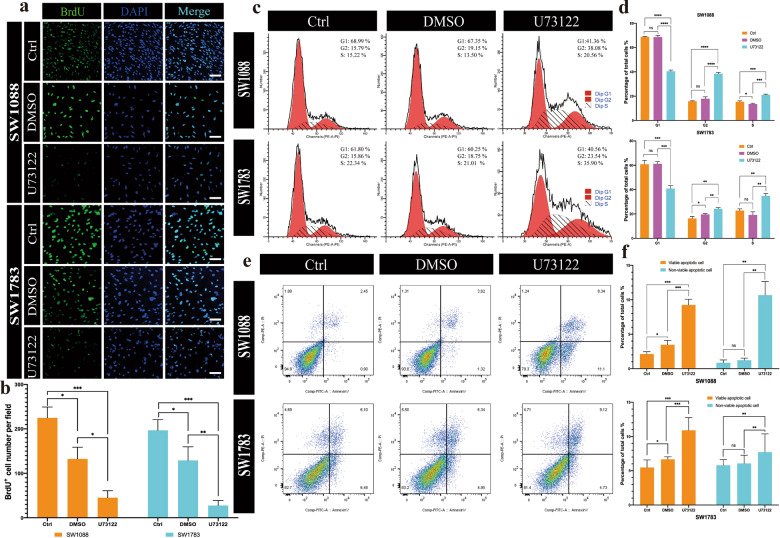
U73122 suppressed the proliferation and cell cycle progression and promoted apoptosis of IDHwt LGG cell lines in vitro. **a** Representative images of the BrdU proliferation assay, indicating the effect of U73122 on SW1088 and SW1783 cells. Scale bar = 30 µm. **b** The statistical results of the BrdU proliferation assay. **c** Representative graphs showing cell cycle distribution. **d** Statistical analysis of the cell cycle distribution (G1, G2, and S) of SW1088 (top) and SW1783 (down) cells in the control, DMSO, and U73122 groups. **e** Representative graphs showing the effect of U73122 on early and late apoptosis by annexin V–PI flow cytometry assays. **f** The statistical results of SW1088 (up) and SW1783 (down) apoptosis assays. ***p < 0.001, **p < 0.01, *p < 0.05. ﻿Data are expressed as the mean ± SD.

We attempted to determine whether a PLCG1-specific inhibitor can treat IDHwt LGG. Although there is no specific small molecule inhibitor of PLCG1, there is a small molecule inhibitor of all PLCs, U73122, which has been reported to potently inhibit receptor-coupled activation of PLC in membranes^27^. For this reason, we hypothesized that U73122 can specifically suppress PLC activity in IDHwt LGG cells, thereby achieving the effect of targeted shutdown. After administration of 5 µM U73122 to SW1088 or SW1783 cells, the tumor cells in the drug group, but not the control and DMSO groups, were significantly affected (Fig. 5), which was consistent with a previous RNAi assay. Moreover, in the U73122 group, the number of BrdU-positive cells decreased 70–80% compared with that in the control group in both the SW1088 and SW1783 groups (Fig. 5a, b). In addition, the percentage of cells in G2 and S phase was 1.5–2 times greater in the U73122 group than in the control or DMSO group in these two cell lines (Fig. 5c, d). Moreover, the apoptotic cells (including viable apoptotic and nonviable apoptotic cells) of both SW1088 and SW1783 cells were distinctly elevated (Fig. 5e, f). These results implied that U73122 could suppress IDHwt LGG cell progression by not only inhibiting their proliferation but also accelerating their apoptosis.

**Fig. 6:**
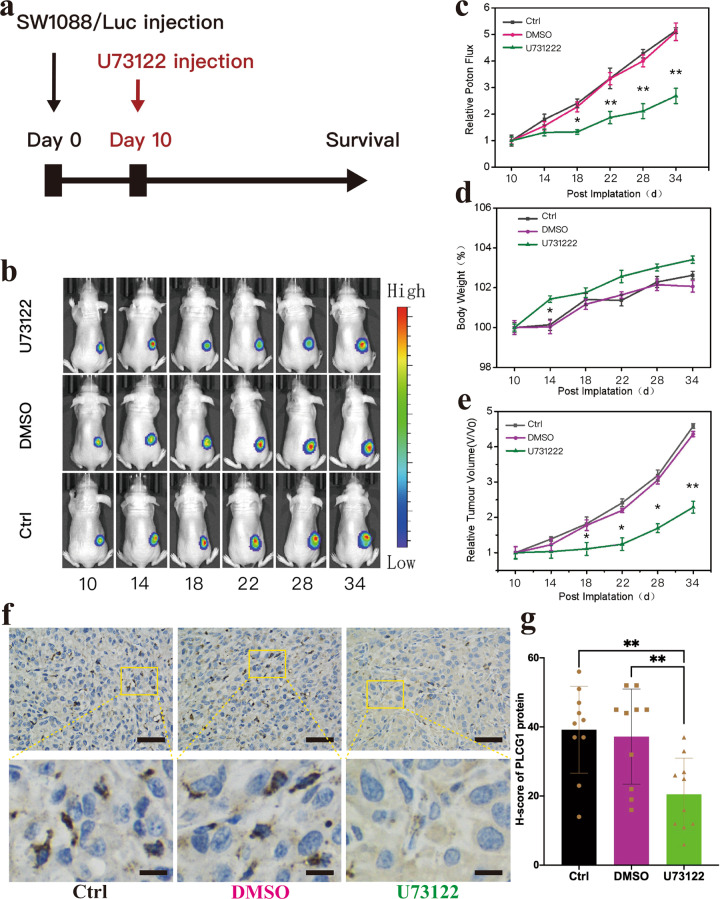
U73122 suppressed tumor growth in mouse subcutaneous xenograft models. **a** Time course of U731222 treatment in the mouse subcutaneous SW1088/Luc xenograft model. **b** Representative images of tumor bioluminescence intensity after SW1088/Luc inoculation after 10, 14, 18, 22, 28, and 34 days. **c** The statistical results of the photon flux of SW1088/Luc xenograft models. **d** The statistical results of the body weights of SW1088/Luc xenograft models. **e** The statistical results of the relative tumor volume of SW1088/Luc xenograft models. **f** Representative IHC image of samples from xenograft model in different groups (upper); Magnification of yellow rectangular area (lower). Scale bar = 50 μm (upper three images) or 10 μm (lower three images). **g** The statistical results of the H-score of PLCG1 protein. **p < 0.01, *p < 0.05. Data are expressed as the mean ± SD.

To further test the anti-tumor effect of U73122 in vivo, we constructed the SW1088/Luc cell line and subcutaneous xenograft models. Ten days after the implantation of SW1088/Luc cells, U73122, DMSO or PBS was retrospectively infused in situ into the mice (Fig. 6a). The administration of U73122 attenuated in vivo tumor growth (Fig. 6b, c). Twenty days after implantation, the tumor volumes of U73122-treated mice were significantly lower than those of the other two groups (Fig. 6e), while their body weights did not differ significantly (Fig. 6d). Next, we ﻿analysed PLCG1 expression in xenograft models. IHC staining revealed clear cytoplasmic localization of PLCG1, and PLCG1 protein in U73122 group was significantly lower than that in control and DMSO groups (Fig. 6f, g), which suggested that U73122 could suppress the synthesis or activity of PLCG1. The survival time was not included because the mice with SW1088 tumor burden lived much longer than those implanted with GBM cell lines, such as U87 and U251. These results demonstrated that U73122 could be a potential and promising PLC-targeted drug for IDHwt LGG in vivo.

## Discussion

LGGs comprise grade II and III gliomas, which account for approximately one-fourth to one-third of gliomas^28,29^. They are diffuse, poorly circumscribed infiltrative tumors frequently localized in the cerebral hemispheres and include oligodendroglioma and astrocytoma. Due to advances in sequencing technologies, LGG can be classified into 3 molecular subtypes: IDHmut +1p19q codeletion, IDHmut only and IDHwt, with each having different clinical manifestations^30^. However, recent publications have shown that LGG is a highly heterogeneous tumor entity^9,10^. Thus, the identification of specific biomarkers for LGG is of great importance, especially for developing potential target chemotherapeutic agents (which have been more thoroughly investigated in glioblastoma WHO IV). In our study, we performed transcriptome profiling for 24 LGGs. Hierarchical clustering revealed the presence of three subtypes among LGGs, with each type exhibiting a distinctive signature. One subtype was characterized by the lack of an IDH mutation (IDH wild-type). Another subtype was characterized by IDH mutation and 1p/19q codeletion, and the remaining subtype harboured an IDH mutation but showed no 1p/19q codeletion. Our results were consistent with the data set generated in the TCGA study^28^.

In our transcriptome profiling data, we identified 302 upregulated genes and 881 downregulated genes in IDHwt LGG. We then focused on overexpressed genes located on chromosome 20 for two reasons. First, gain of chromosome 20 is the most frequent genomic aberration, followed by gain of chromosome 7 and loss of chromosome 10, in IDHwt LGG^31,32^. Second, gain of chromosome 20 is exclusively found in IDHwt LGG^30^. Further bioinformatics analysis identified PLCG1 as an essential oncogene. PLCG1 has been reported to be a multifunctional protein abundant in most malignant tumors, including liver, lung, and prostate cancer^18,19,23,24^. Its expression is strongly linked with poor outcome^19,24,33^. To the best of our knowledge, our paper is the first study to report that a high expression level of PLCG1 correlated with shorter survival in IDHwt LGG. We also performed survival analysis of the PLCG1 expression level in IDHwt glioblastoma (data not shown) and found that increased expression of PLCG1 can act as an indicator of shorter survival but with no statistical significance, which indicated the distinct biological effects of PLCG1 on IDHwt LGG. Meanwhile, gene upregulation is often accompanied by gene amplification^34–36^ in gliomas; for example, EGFR expression is strongly affected by EGFR amplification^37^. Therefore, we investigated whether the mechanism of PLCG1 dysregulation in IDHwt LGG correlated with PLCG1 amplification. We found a high ratio of PLCG amplification in IDHwt LGG, especially in cases with high PLCG1 expression levels. At the same time, we examined somatic PLCG1 mutations in 2 IDHwt LGGs by next-generation sequencing. Sanger sequencing confirmed that one IDHwt LGG harboured a missense mutation in exon 19 of PLCG1 (Fig. S5). The mutation is located on the highly evolutionarily conserved C-terminal SH2 domain, which is involved in autoinhibition. In this LGG, the mutation resulted in a nucleotide change from A to G at position 2248 (NM_002660) and an amino acid change from methionine (M) to valine (V) at position 750 (NP_002651.2). This evidence showed that both genomic and genetic alterations of PLCG1 contribute to the oncogenesis of IDHwt LGG.

In addition, PLCG1 has multiple biological roles. This is implied by its distribution in multiple cellular compartments and interactions with various molecules and second messengers, which in turn result in positive effects on cell growth among various cancers^19,24,33,38^. However, no relevant PLCG1 studies have focused on LGGs. In our IDHwt LGG samples, we showed that decreased PLCG1 expression suppressed tumor proliferation, migration and invasion and promoted apoptosis owing to a disturbance of the essential functions of PLCG1, such as protein secretion, MYC targets, mitotic spindle, the G2/M checkpoint, and E2F targets. These results suggested that PLCG1, as a hub of many physiological processes, plays an indispensable role in various tumors, including IDHwt LGGs.

U73122 has been reported to be effective in inhibiting PLC in prostate cancer, lung cancer and breast cancer^35,36,39^. Here, we reported that U73122 could inhibit IDHwt LGGs progression by not only suppressing cell proliferation but also stimulating apoptosis. In addition, the in vivo results further verified the effect of U73122 on IDHwt LGGs. Taken together, our findings demonstrated that U73122 might be a potential and novel PLC-targeted therapy for IDHwt LGG patients in future clinical practice.

Indeed, there were several limitations in this research work. RNA sequencing, IHC and FISH were performed in a quite smaller sample size, especially the genomic and genetic alterations in PLCG1 dysregulation should be further investigated. Meanwhile, more in-depth studies are needed to explore the specific signaling pathways of PLCG1 related to the tumor malignant transformation in IDHwt LGGs. These studies will be emphasized in the future work.

Our study showed the highly heterogeneity of LGGs at the transcriptome level and identified PLCG1 as a novel molecular biomarker of IDHwt LGGs. To the best of our knowledge, this is the first report to verify the essential role of PLCG1 in the biological process of IDHwt LGGs, notably in facilitating cancer proliferation and adaptation. More importantly, PLCG1 is associated with malignant progression of IDHwt LGGs and indicates dismal clinical outcomes in IDHwt LGG patients. Depletion of PLCG1 by RNAi or PLC-targeted drugs could be a potential therapy for treating IDHwt LGG treatment.

## Supplementary information

All Supplemental Materials

## Data Availability

The TCGA and CGGA data are openly available in https://www.cancer.gov/about-nci/organization/ccg/research/structural-genomics/tcga/ and http://www.cgga.org.cn/. Other data used to support the findings of this study are available from the corresponding author upon request.
