# Supplementary material for: Phospholipase Cγ1 (PLCG1) overexpression is associated with tumor growth and poor survival in IDH wild-type lower-grade gliomas in adult patients
Source: Lab Invest. 2021 Oct 25;102(2):143–53. doi: 10.1038/s41374-021-00682-7 (PMC8784314; doi:10.1038/s41374-021-00682-7)
Supplement: Supplementary file 1 — All Supplemental Materials [file 41374_2021_682_MOESM1_ESM.pdf]

Supplemental Materials

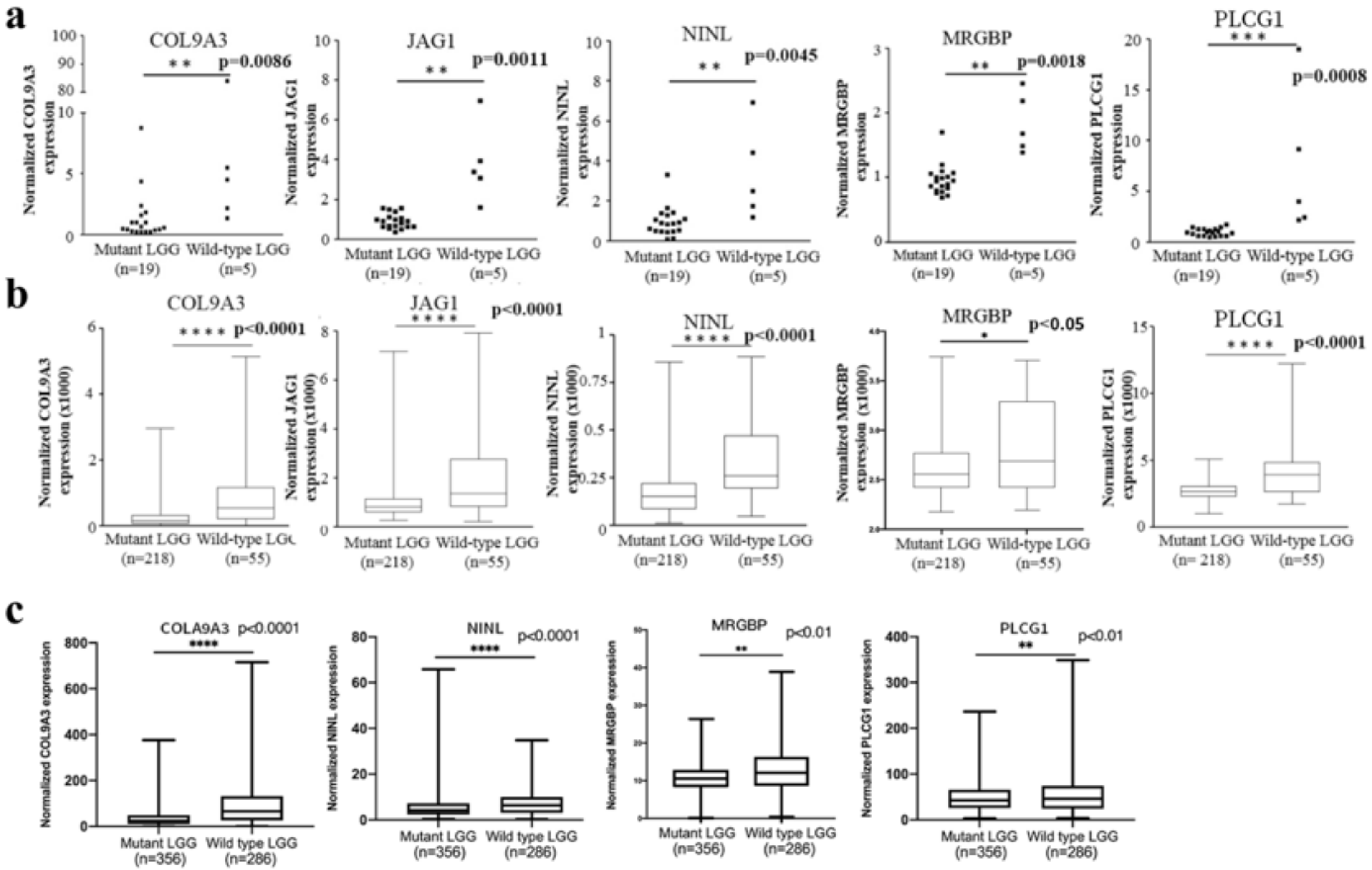
a
COL9A3
p=0.0086
Normalized COL9A3 expression
JAG1
p=0.0011
Normalized JAG1 expression
NINL
p=0.0045
Normalized NINL expression
MRGBP
p=0.0018
Normalized MRGBP expression
PLCG1
p=0.0008
Normalized PLCG1 expression
Mutant LGG (n=19)
Wild-type LGG (n=5)
b
COL9A3
p<0.0001
Normalized COL9A3 expression (x1000)
JAG1
p<0.0001
Normalized JAG1 expression (x1000)
NINL
p<0.0001
Normalized NINL expression (x1000)
MRGBP
p<0.05
Normalized MRGBP expression (x1000)
PLCG1
p<0.0001
Normalized PLCG1 expression (x1000)
Mutant LGG (n=218)
Wild-type LGG (n=55)
c
COLA9A3 p<0.0001
Normalized COL9A3 expression
NINL p<0.0001
Normalized NINL expression
MRGBP p<0.01
Normalized MRGBP expression
PLCG1 p<0.01
Normalized PLCG1 expression
Mutant LGG (n=356)
Wild type LGG (n=286)

Supplemental Figure 1 The COL9A3, JAG1, NINL, MRGBP and PLCG1 expression levels in three different cohorts.

a The expression of these five genes on chromosome 20 in IDHwt group were significantly higher than that in IDHmut group in our RNA-seq cohort.

b The expression of these five genes on chromosome 20 in IDHwt group were significantly higher than that in IDHmut group in TCGA_LGG cohort.

c The expression of these five genes on chromosome 20 in IDHwt group were significantly higher than that in IDHmut group in CGGA_LGG cohort.

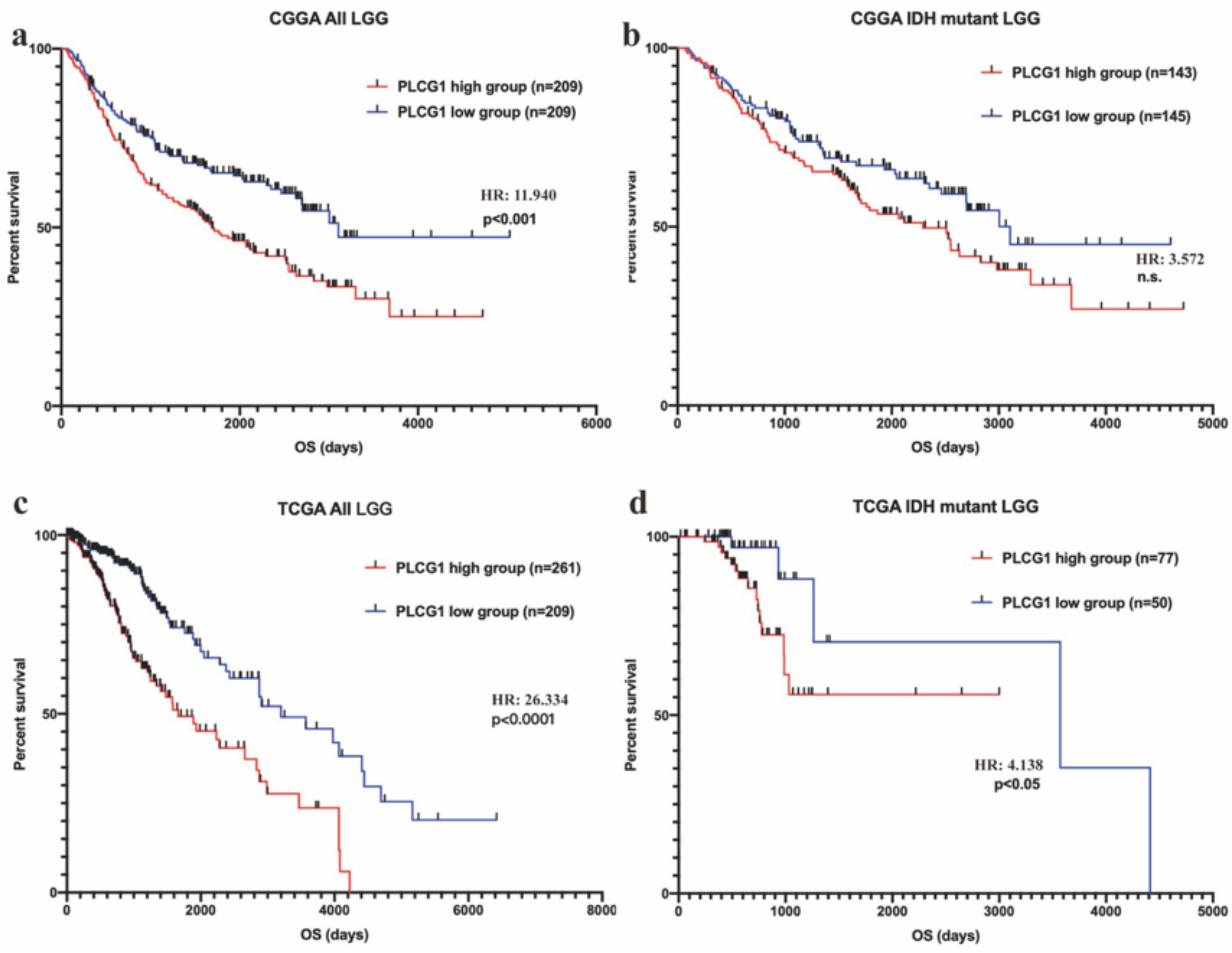
a
CGGA All LGG
PLCG1 high group (n=209)
PLCG1 low group (n=209)
HR: 11.940
p<0.001
Percent survival
OS (days)
b
CGGA IDH mutant LGG
PLCG1 high group (n=143)
PLCG1 low group (n=145)
HR: 3.572
n.s.
OS (days)
c
TCGA All LGG
PLCG1 high group (n=261)
PLCG1 low group (n=209)
HR: 26.334
p<0.0001
Percent survival
OS (days)
d
TCGA IDH mutant LGG
PLCG1 high group (n=77)
PLCG1 low group (n=50)
HR: 4.138
p<0.05
Percent survival
OS (days)

Supplemental Figure 2 Kaplan-Meier survival analysis of PLCG1 in LGGs from public databases

a Kaplan-Meier survival analysis of PLCG1 in all LGG patients from the CGGA_LGG database.

b Kaplan-Meier survival analysis of PLCG1 in IM patients from the CGGA_LGG database.

c Kaplan-Meier survival analysis of PLCG1 in all LGG patients from the TCGA_LGG database.

d Kaplan-Meier survival analysis of PLCG1 in IM patients from the TCGA_LGG database.

***p<0.001, **p<0.01, *p<0.05. Data are expressed as the mean ± SD.

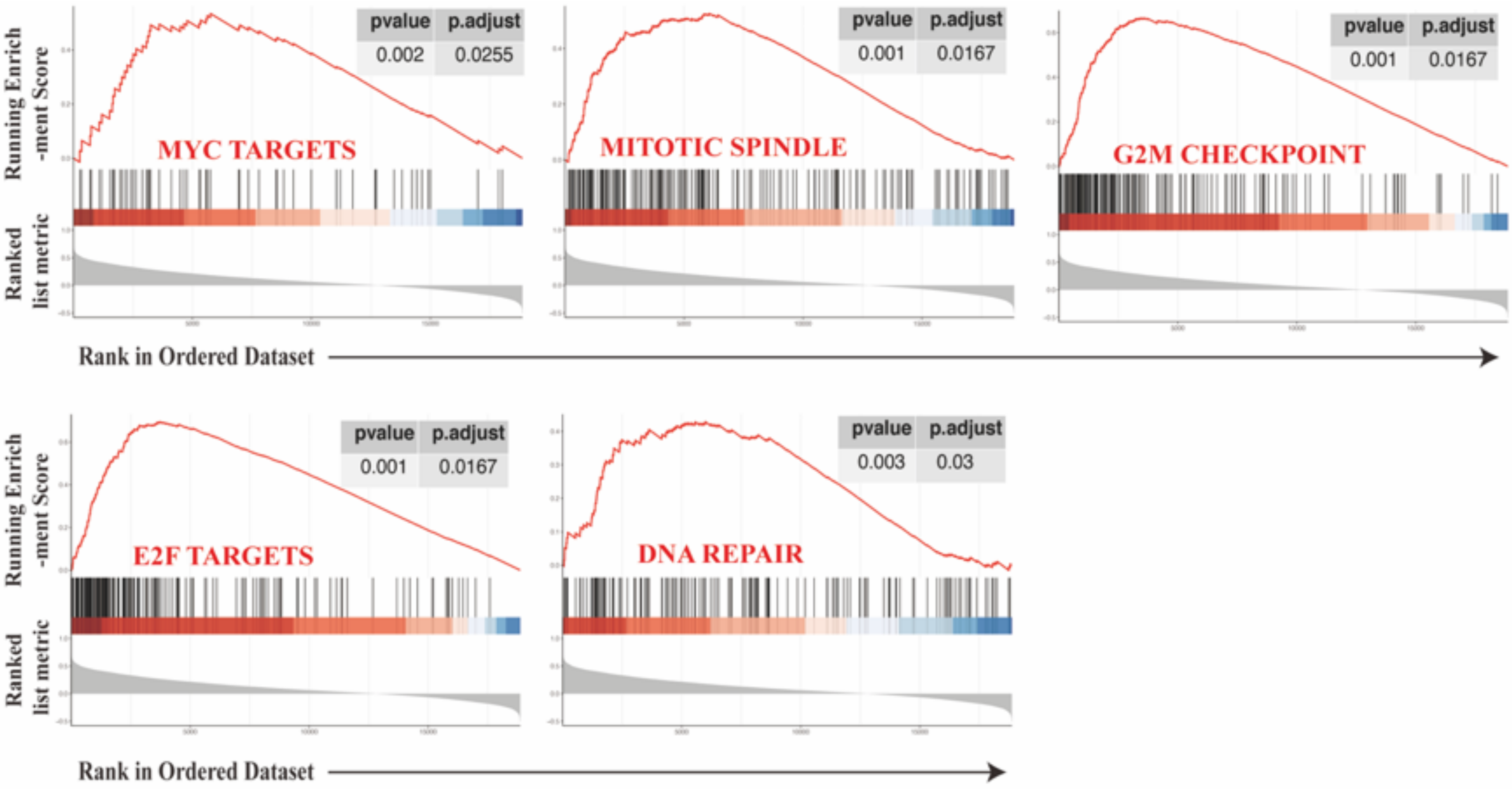

Supplemental Figure 3 Enrichment plots of GSEA using the TCGA gene expression profiles of IDHwt LGG.

Enrichment plots of GSEA using the CGGA gene expression profiles of IDHwt LGG indicated the top 5 functions in which PLCG1 is involved: MYC targets, the mitotic spindle, G2/M checkpoint, E2F targets and DNA repair.

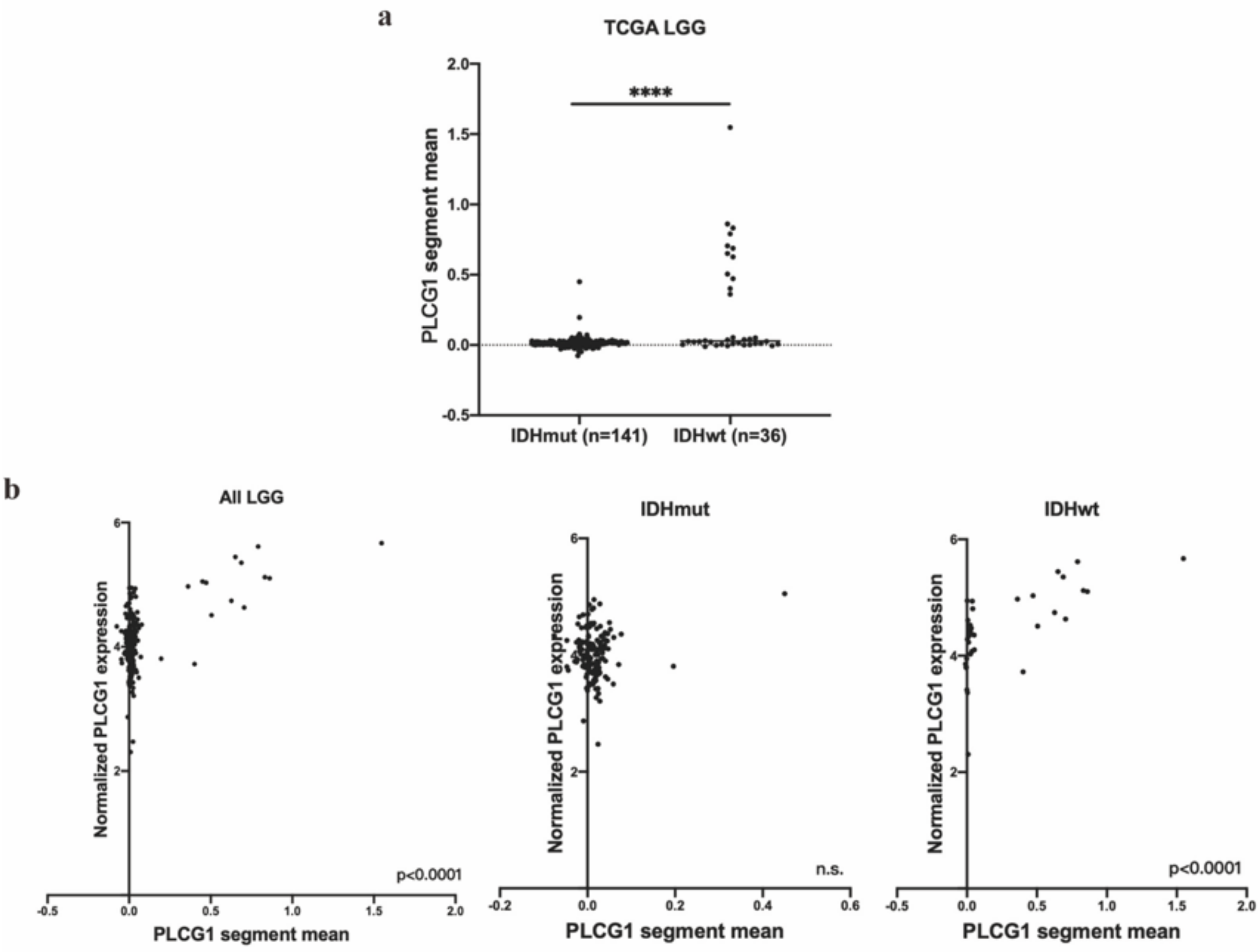
a
TCGA LGG
PLCG1 segment mean
****
2.0
1.5
1.0
0.5
0.0
-0.5
IDHmut (n=141)
IDHwt (n=36)
b
All LGG
IDHmut
IDHwt
Normalized PLCG1 expression
PLCG1 segment mean
p<0.0001
n.s.
p<0.0001

Supplemental Figure 4 PLCG1 copy number gain/amplification difference between IDHwt and IDHmut LGGs

a PLCG1 copy number gain/amplification was significantly higher in IDHwt group than IDHmut group.

b PLCG1 copy number gain/amplification was strongly correlated with PLCG1 mRNA level in IDHwt group but not in IDHmut group.

****p<0.0001, ***p<0.001, **p<0.01, *p<0.05. Data are expressed as the mean ± SD.

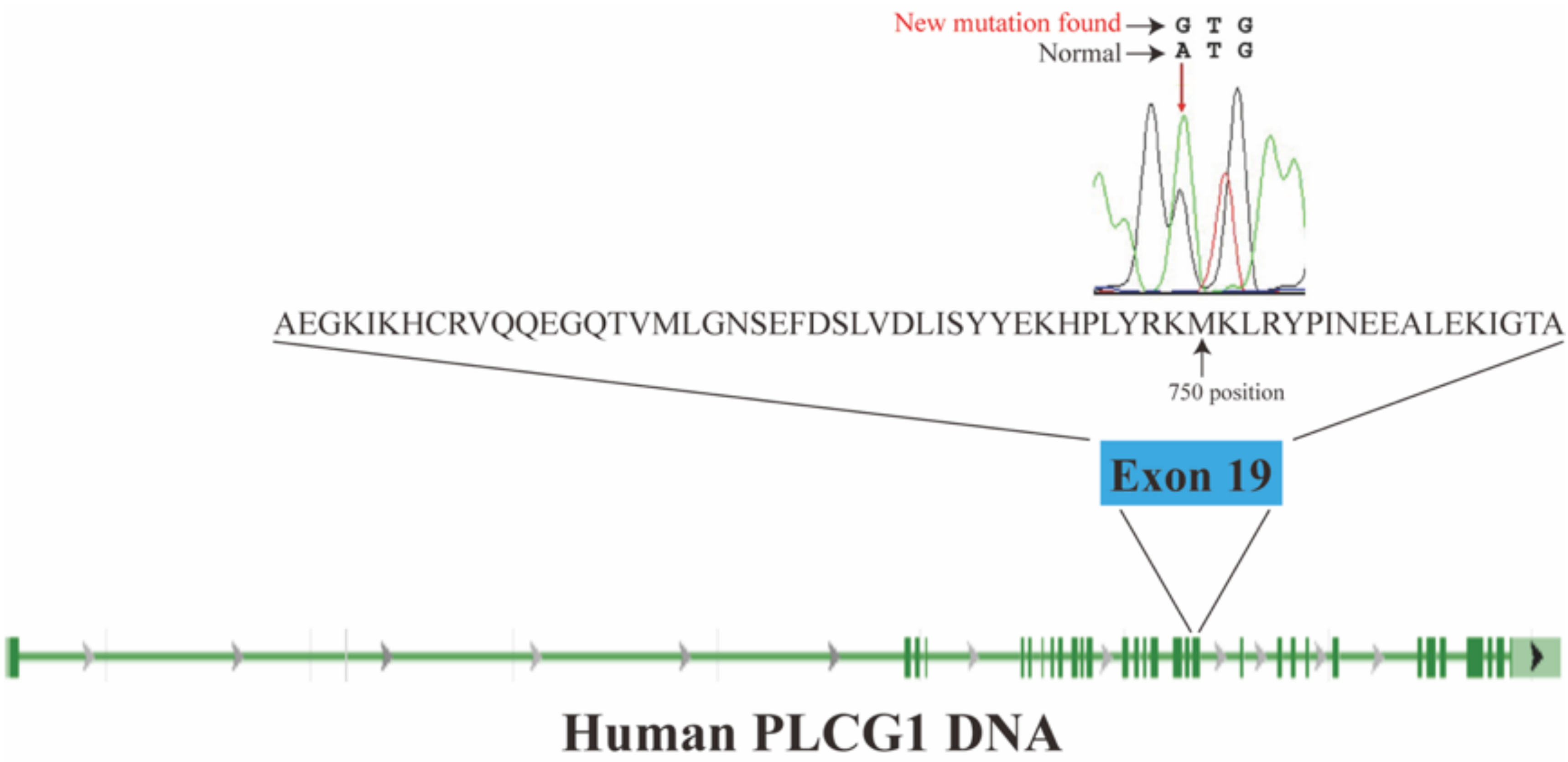

Supplemental Figure 5 Identification of a novel PLCG1 mutation in IDH1/2 wild-type LGG.

Next-Generation Sequencing confirmed that an IDHwt LGG harboured a missense mutation in exon 19 of PLCG1.

Supplemental Table 1 Demographic and follow-up information of patients in Huashan hospital

Case number	gender	Age	Histology	WHO grade	IDH 1/2	PLCG1 mRNA level (relative to GAPDH)	Survival (death=1; alive=0)	OS (months)
HS001708	F	39	Astrocytoma	III	wt	-5.536787987	1	25
HS001348	M	66	Oligodendroglioma	III	wt	-5.103207588	1	24
HS001948	F	46	Astrocytoma	II	wt	-3.564608574	1	12
HS001280	M	14	Astrocytoma	III	wt	-3.250869751	0	NA
HS000233	F	39	Astrocytoma	II	wt	-2.262991905	1	66
HS001381	M	32	Astrocytoma	II	wt	-1.962715149	1	39
HS001682	F	82	Astrocytoma	III	wt	-1.786524773	1	18
HS000189	F	41	Astrocytoma	II	wt	-1.705540657	1	28
HS001126	M	48	Astrocytoma	II	wt	-1.651386261	1	33
HS000274	M	64	Astrocytoma	II	wt	-1.438883781	1	68
HS002481	M	58	Astrocytoma	II	wt	-1.391281128	0	NA
HS001520	F	57	Astrocytoma	II	wt	-1.360155106	1	30
HS000320	M	39	Astrocytoma	II	wt	-1.200849533	1	23
HS002592	F	49	Astrocytoma	III	wt	-1.148181915	1	20
HS000442	M	71	Astrocytoma	II	wt	-1.124094009	1	38
HS001874	F	58	Astrocytoma	III	wt	-0.927822113	0	NA
HS002188	F	66	Astrocytoma	III	wt	-0.919172287	1	21
HS001679	M	32	Astrocytoma	III	wt	-0.779790878	1	67
HS000440	M	36	Astrocytoma	II	wt	-0.748829842	1	31
HS001042	F	51	Oligodendroglioma	II	wt	-0.667085648	1	33
HS000437	F	63	Oligodendroglioma	II	wt	-0.440056801	1	34

HS001903	F	19	pleomorphic xanthoastrocytoma	II	wt	-0.35619545	0	NA
HS000610	M	43	Astrocytoma	III	wt	-0.300171852	1	18
HS001439	M	57	Astrocytoma	III	wt	-0.250995636	1	37
HS002079	F	52	Astrocytoma	III	wt	-0.244243622	1	NA
HS000855	F	58	Astrocytoma	III	wt	-0.233705521	1	26
HS001524	M	46	Astrocytoma	II	wt	-0.215697289	1	12
HS002075	M	54	Oligodendroglioma	II	wt	-0.16984272	0	NA
HS001258	F	71	Astrocytoma	III	wt	-0.168447495	1	29
HS000617	M	49	Astrocytoma	III	wt	-0.152105331	1	52
HS000681	M	77	Oligodendroglioma	II	wt	-0.139734268	1	35
HS000195	M	58	Oligodendroglioma	II	wt	-0.131448746	1	95
HS001815	F	46	Astrocytoma	III	wt	-0.123736382	1	18
HS000693	M	67	Astrocytoma	III	wt	-0.078547478	1	31
HS001108	M	48	Astrocytoma	III	wt	-0.040850639	1	43
HS002609	M	15	Astrocytoma	II	wt	-0.03126812	1	28
HS001269	M	44	Astrocytoma	II	wt	0.021756172	1	61
HS001541	M	29	Astrocytoma	III	wt	0.134871483	1	67
HS000302	M	62	Astrocytoma	III	wt	0.192778587	1	103
HS001071	M	67	Astrocytoma	III	wt	0.230211258	1	22
HS000330	M	70	Astrocytoma	III	wt	0.409109116	1	33
HS001237	F	69	Astrocytoma	II	wt	0.488924026	1	25
HS001460	F	33	Astrocytoma	III	wt	0.494145393	0	NA
HS001895	M	51	Astrocytoma	III	wt	0.502015114	1	17
HS000926	M	25	Astrocytoma	III	wt	0.609566689	1	16

HS002027	M	64	Astrocytoma	III	wt	0.650065422	1	17
HS001422	F	50	Astrocytoma	III	wt	0.827513695	1	17
HS000473	M	74	Oligodendroglioma	III	wt	0.874873161	1	14
HS001414	M	49	Astrocytoma	III	wt	0.895154953	1	26
HS002336	M	29	Astrocytoma	II	wt	0.912822723	0	NA
HS000913	M	66	Astrocytoma	III	wt	0.937279701	1	12
HS000725	F	43	Astrocytoma	III	wt	1.091553688	1	21
HS001940	F	61	Oligodendroglioma	III	wt	1.115790367	1	20
HS002405	M	55	Astrocytoma	III	wt	1.125824928	1	27
HS001615	M	53	Astrocytoma	III	wt	1.131703377	1	24
HS002264	F	37	Astrocytoma	III	wt	1.138157845	1	10
HS001316	F	38	Oligodendroglioma	III	wt	1.14854908	1	30
HS000772	M	58	Astrocytoma	III	wt	1.189653397	1	12
HS001344	M	68	Astrocytoma	III	wt	1.233850479	1	2
HS000738	M	73	Oligodendroglioma	III	wt	1.234389305	1	7
HS000732	M	69	Astrocytoma	III	wt	1.23916626	1	3
HS002139	F	18	Astrocytoma	II	wt	1.460699081	1	28
HS002523	F	37	Oligodendroglioma	II	wt	1.519686699	1	22
HS000673	M	53	Astrocytoma	III	wt	1.556434631	1	22
HS001493	M	39	Astrocytoma	II	wt	1.567727089	1	49
HS001240	M	59	Astrocytoma	III	wt	1.609639168	1	19
HS000538	F	56	Astrocytoma	II	wt	1.680265427	1	37
HS001352	M	69	Astrocytoma	II	wt	1.706268311	1	41
HS000932	M	30	Oligodendroglioma	II	wt	1.828027725	1	10
HS002057	F	54	Astrocytoma	III	wt	1.82954216	1	28

HS001368	M	75	Astrocytoma	II	wt	1.840145111	1	14
HS002053	F	64	Astrocytoma	III	wt	1.869277	1	8
HS002250	M	65	Astrocytoma	III	wt	1.915079117	1	35
HS002031	M	48	Astrocytoma	II	wt	1.978390694	1	17
HS000828	M	53	Astrocytoma	III	wt	1.991726875	1	16
HS002010	M	77	Astrocytoma	III	wt	2.097821236	1	12
HS000670	F	72	Astrocytoma	III	wt	2.098917007	1	27
HS001054	F	54	Astrocytoma	II	wt	2.108097076	1	6
HS001510	M	65	Oligodendroglioma	III	wt	2.137231827	1	83
HS002426	M	65	Astrocytoma	II	wt	2.147414207	1	11
HS001823	M	58	Astrocytoma	III	wt	2.16795826	1	4
HS000626	M	74	Astrocytoma	II	wt	2.233940125	1	38
HS001457	F	59	Astrocytoma	III	wt	2.238453865	1	13
HS001406	M	22	Astrocytoma	II	wt	2.327997208	0	NA
HS002065	M	38	Astrocytoma	III	wt	2.340386391	1	6
HS000598	M	32	Astrocytoma	II	wt	2.34677887	1	4
HS002615	F	48	Astrocytoma	II	wt	2.379321098	1	17
HS000599	M	59	Oligodendroglioma	III	wt	2.576972008	1	16
HS001115	F	68	Astrocytoma	III	wt	2.598757744	1	64
HS002263	M	64	Oligodendroglioma	III	wt	2.693796158	1	12
HS002349	M	37	Oligodendroglioma	III	wt	2.771742821	1	29
HS001533	F	54	Astrocytoma	III	wt	2.83595562	1	35
HS002375	M	51	Oligodendroglioma	II	wt	2.991323471	0	NA
HS001719	M	54	Astrocytoma	II	wt	3.092228889	1	18
HS001489	F	46	Oligodendroglioma	III	wt	3.132577896	1	69

HS002095	M	57	Astrocytoma	II	wt	3.150182724	1	16
HS002207	F	65	Oligodendroglioma	III	wt	3.193610191	1	38
HS000770	M	83	Astrocytoma	III	wt	3.346335411	1	6
HS001313	F	58	Astrocytoma	II	wt	3.364608765	0	NA
HS001023	M	65	Astrocytoma	III	wt	3.401069641	1	23
HS001516	M	51	Astrocytoma	III	wt	3.455292702	1	7
HS002379	M	63	Oligodendroglioma	III	wt	3.47376442	1	26
HS000724	M	67	Astrocytoma	III	wt	3.777253151	1	15
HS001784	M	70	Oligodendroglioma	III	wt	3.874172211	1	10
HS000502	M	67	Oligodendroglioma	III	wt	3.879079819	1	19
HS001585	M	70	Astrocytoma	III	wt	4.003928185	1	2
HS002579	M	53	Astrocytoma	III	wt	4.056534767	1	31
HS001907	M	58	Astrocytoma	III	wt	4.542236328	1	20
HS000481	M	47	Astrocytoma	III	wt	4.840776443	1	39
HS000774	M	55	Astrocytoma	III	wt	4.945576668	0	15
HS000218	M	70	Oligodendroglioma	III	wt	5.03045845	1	11
HS002255	M	49	Astrocytoma	III	wt	5.137647629	1	11
HS001386	M	22	Astrocytoma	III	wt	5.209362984	1	13
HS001709	F	67	Astrocytoma	III	wt	5.490337372	1	15
HS002617	M	55	Astrocytoma	III	wt	5.764357567	1	11
HS001848	F	48	Astrocytoma	III	wt	6.090341568	1	20
HS001924	M	36	Astrocytoma	III	wt	6.126196861	1	18
HS001925	M	49	Astrocytoma	III	wt	6.206400871	1	49
HS001405	M	62	Astrocytoma	II	wt	6.581041336	1	10
HS002096	F	35	Astrocytoma	II	wt	6.593013763	1	10

HS001934	M	44	Oligodendroglioma	III	wt	6.674445152	1	50
HS001274	M	71	Astrocytoma	II	wt	7.081605911	1	16
HS001326	M	63	Astrocytoma	II	wt	7.169106483	1	7
HS001796	M	65	Astrocytoma	III	wt	7.696310997	1	28
HS001288	F	59	Astrocytoma	III	wt	8.614797592	1	13

Supplemental Table 2 QPCR, IHC and FISH results of PLCG1 in representative patients in Huashan hospital

Case number	gender	Age	Histology	WHO grade	IDH 1/2	PLCG1 IHC score	PLCG1 positive?	PLCG1 amplification (Yes/no)	PLCG1 expression (relative to TBP)
G2390	M	33	oligodendroglioma	II	mutant	0	No	No	0.420879261
G2125	M	14	astrocytoma	III	wt	4	Yes	No	1.116512962
G2199	M	58	astrocytoma	III	mutant	0	No	No	1.136161816
G2304	M	75	oligoastrocytomas	III	mutant	2	No	No	1.193931415
G1974	F	35	oligodendroglioma	III	mutant	2	No	No	1.328378858
G1797	M	58	astrocytoma grade	II	mutant	0	No	No	1.345367209
G2020	M	32	astrocytoma	II	mutant	2	No	No	1.891241153
G2075	F	54	oligodendroglioma	II	wt	2	No	No	1.926818602
G2389	M	62	astrocytoma	II	mutant	0	No	No	1.995845438
G1967	F	49	astrocytoma	II	mutant	3	No	No	2.086377187
G2197	M	37	astrocytoma	II	mutant	0	No	No	2.249077435
G2220	F	63	astrocytoma	III	mutant	0	No	Yes	2.866585546
G2207	M	42	astrocytoma	III	wt	4	Yes	Yes	2.892552515
G2064	M	53	astrocytoma	II	mutant	0	No	Yes	3.113725232
G2201	M	47	astrocytoma	II	wt	4	Yes	No	3.168207148
G2320	M	26	astrocytoma	III	wt	9	Yes	Yes	3.658255909
G2212	M	27	astrocytoma	III	wt	2	No	No	4.060526114
G1803	F	58	astrocytoma	III	wt	3	No	No	4.416099367
G1697	M	68	astrocytoma	III	wt	6	Yes	Yes	4.809876996
G2149	F	38	oligodendroglioma	II	wt	6	Yes	Yes	19.23950798
G2163	M	51	oligodendroglioma	II	wt	4	Yes	Yes	26.90246878

G2182	M	36	astrocytoma	II	wt	6	Yes	Yes	27.43603317

Supplemental Table 3 PLCG1 siRNA sequence

	Sense (5’-3’)	Antisense (5’-3’)
PLCG1-Homo-336	CCAUUGACAUUCGUGAAAUTT	AUUUCACGAAUGUCAAUGGTT
PLCG1-Homo-1303	GCACACCAUCAAGGAGCAUTT	AUGCUCCUUGAUGGUGUGCTT
PLCG1-Homo-1384	GCAGAGAAACAUGGCCCAATT	UUGGGCCAUGUUUCUCUGCTT
